# Albumin-Based Indices and Hematologic Ratios as Predictors of Short-Term Response to Anti-TNF Therapy in Crohn’s Disease: A Single-Center Retrospective Study

**DOI:** 10.3390/life16071126

**Published:** 2026-07-07

**Authors:** Aleksandra Sobolewska-Włodarczyk, Filip Romaniuk, Dominika Bałuch, Anita Gąsiorowska

**Affiliations:** Department of Gastroenterology, Medical University of Łódź, 90-149 Lodz, Poland

**Keywords:** Crohn’s disease, infliximab, adalimumab, early response prediction, albumin, CALLY, CRP/albumin ratio

## Abstract

Background Biological agents such as infliximab [IFX] and adalimumab [ADA] are employed in the treatment of severe Crohn’s disease [CD], especially in cases refractory to conventional therapies. This study assessed the predictive value of simple serum-based markers in terms of early response to anti-tumor necrosis factor [anti-TNF] biologics. Methods:A total of 55 adult patients who had undergone 57 individual courses of either IFX or ADA at the Department of Gastroenterology of the Medical University of Łódź were retrospectively evaluated: 33 males and 22 females, aged 18–70 years. Serum parameters were assessed upon initiation of biological therapy and at the 14th week treatment response assessment juncture. Results: A total of 50 of the 57 (87.7%) assessed courses presented with treatment response upon week 14. Intergroup comparison revealed a statistically significant discrepancy in albumin concentration between the responder and non-responder groups: 38.4 g/L (35–41) vs. 28.4 g/L (26.7–30.1) (*p* < 0.05). Similar results were observed upon division of the total group in regard to prior biological agent exposure—bionaïves and biopositives. Further receiver operating characteristic [ROC] analysis revealed either strong or excellent discriminatory performance in regard to early response in the total and biopositive groups of mean albumin concentration (AUC: 0.93, *p* < 0.05; 1.0, *p* < 0.05), CRP–albumin–lymphocyte [CALLY] (AUC: 0.81, *p* < 0.05; 0.88, *p* < 0.05), CRP/albumin ratio (AUC: 0.83, *p* < 0.05; 0.88, *p* < 0.05), and mean platelet volume [MPV] and red blood cell distribution width [RDW] in the bionaïve group (AUC: 0.87, *p* < 0.05) (AUC: 0.88, *p* < 0.05), respectively. Conclusions: Simple, readily available serum-derived markers appear promising in assessing the likelihood of early anti-TNF response in CD patients. Albumin and albumin-derived parameters especially demonstrated considerable potential in this regard.

## 1. Introduction

Crohn’s disease (CD) is a chronic, progressive inflammatory bowel disease in which up to one-third of patients do not respond to anti-tumor necrosis factor (anti-TNF) induction therapy and up to half of initial responders lose response over time [1,2]. The etiology of CD is presently unknown [3]. There are, however, numerous hypotheses regarding its origin, with most emphasizing a mixture of environmental, geographical and genetic factors [4,5]. Several studies have also linked the incidence of IBD to smoking tobacco as well as to disruptions in the gut microbiota [6].

Identifying simple, low-cost biomarkers that predict treatment response is therefore a major unmet need. Serum albumin and albumin-based indices, such as the C-reactive protein/albumin ratio and the CALLY index, integrate inflammatory and nutritional status and have emerged as promising biomarkers in IBD and other immune-mediated diseases [7,8,9]. Similarly, blood cell-derived indices, including mean platelet volume (MPV) and red cell distribution width (RDW), have been linked to disease activity in Crohn’s disease, but their role as predictors of biologic response remains unclear [10,11]. In this single-center retrospective study, we aimed to evaluate whether baseline albumin, CALLY index, CRP/albumin ratio and hematologic indices (MPV, RDW and others) are associated with clinical response at week 14 in CD patients treated with infliximab (IFX) or adalimumab (ADA).

## 2. Materials and Methods

This study was conducted in a retrospective manner in 55 adult patients aged 18–70 years with Crohn’s disease who were qualified for biological treatment with either IFX or ADA and completed at least 14 weeks of treatment at the Department of Gastroenterology of the Medical University of Lodz. This study was assessed by the Medical University of Lodz Bioethics Committee, which confirmed this study did not meet the criteria of medical experiment under applicable national regulations; therefore, a formal approval decision was not issued. This study was conducted in accordance with the Declaration of Helsinki and appropriate data-protection requirements.

A total of 57 courses of treatment were assessed. Analyzed parameters included Crohn’s Disease Activity Index [CDAI], blood serum analysis parameters, sociodemographic factors such as age, sex, disease duration, and body mass, as well as previous exposure to biological treatment.

The patients’ CD activity was gauged with the CDAI—a predominantly clinical symptom-based scoring system employed in the assessment of CD severity. It includes parameters such as weight, ideal body weight, sex, total number of soft/liquid stools in the last 7 days, abdominal pain, general well-being, anti-diarrhea drug use, presence of abdominal mass, hematocrit and presence of extraintestinal manifestations. CDAI scores can range from 0 to 600: a score of <150 indicates remission, 150–219 indicates mildly active disease, 220–450 indicates moderately active disease and >450 indicates severe disease. A margin of >70 or a 25% CDAI reduction at the 14th week of treatment is considered clinical response and validates further treatment. As such, this study’s endpoint should be considered clinical response gauged with CDAI. The eligibility criteria for biological therapy were defined by the National Drug Program, a state-funded reimbursement program administered by the National Health Fund, Poland’s national healthcare financing institution.

The enrollment criteria for biological therapy included current diagnosis of CD according to the diagnostic guidelines published by the European Crohn’s and Colitis Organization [ECCO] and either CD exacerbation of CDAI > 220 with concurrent lack of response to corticosteroids and other immunosuppressants, the development of perianal fistulae refractory to antibiotics/immunosuppressive treatment or surgery—regardless of CDAI score or a Rutgeerts score of ≥2 in patients who had undergone resective surgery. All qualified patients had been cleared of any underlying malignancy, tuberculosis, human immunodeficiency virus or hepatitis type B/C infection.

From an initial total of 64 patients who had undergone 69 individual courses of therapy, 12 were excluded. Exclusion criteria included cessation of treatment for unknown reasons (2 courses), anaphylaxis (2 courses), initiation of treatment at a pediatric facility (3 courses), and change in administered treatment before the 14th week mark (5 courses).

Upon inclusion, the body mass index [BMI] was calculated based on the height and weight measurements in each patient: BMI = weight in kilograms/height in m^2^. The normal range was considered 20–25 kg/m^2^, with obesity >30 kg/m^2^, overweight 25–30 kg/m^2^, borderline underweight 18.5–20 kg/m^2^, and underweight <18.5 kg/m^2^.

Furthermore, serum markers, such as platelet-to-lymphocyte ratio (platelet count/lymphocyte count [PLR]), neutrophil-to-lymphocyte ratio (neutrophile count/lymphocyte count [NLR]), monocyte-to-lymphocyte ratio (monocyte count/lymphocyte count [MLR]), albumin/CRP ratio (albumin concentration/CRP concentration), C-reactive protein–albumin–lymphocyte index (albumin concentration × lymphocyte count)/(CRP concentration × 10,000) [CALLY]), systemic immune-inflammation index (platelet count × neutrophil count)/lymphocyte count [SII]), mean platelet volume [MPV], and red blood cell distribution width [RDW], were assessed based on test results at the time of qualifying for biological therapy and at the week 14 response assessment checkpoint.

From all patients, an adequate amount of venous blood was taken into standardized tubes containing ethylenediaminetetraacetic acid. Serum levels of LYM, PLT, NEU, MON, MPV, RDW, CRP and albumin were analyzed. All samples were collected before initial administration of treatment. The normal reference ranges were: 1000–5000/μL for LYM, 150,000–400,000/µL for PLT, 1800–8000/μL for NEU, 200–800/μL for MON, 7.5–10.5 fL for MPV, 11.5–14.5% for RDW, 0.08–3.1 mg/L for CRP and 35–50 g/L for albumin. Assessed parameters were analyzed with the Sysmex XN - 2000 and XN - 1000 (Sysmex, Kakogawa, Hyogo Prefecture, Japan) for whole blood count and the Beckman Coulter AU5800 and DXC700AU (Beckman Coulter, Brea, CA, USA) for biochemical analysis.

Statistical analysis of acquired data was performed with the statistical package Statistica 13.3.721.0 [TIBCO Software Inc., Tulsa, OK, USA].

Assessed biomarkers and parameters were selected a priori on the basis of clinical relevance and previous evidence. As the analyses were hypothesis driven and exploratory in nature, *p*-values were not adjusted for multiple comparisons and therefore should be interpreted cautiously.

The quantitative variables are presented as arithmetical mean and standard deviation or the median and interquartile range depending on normal or abnormal distribution of variables. The normality of distribution was assessed with the Shapiro–Wilk test. Categorical variables are presented as numbers and percentages.

The intergroup differences for the quantitative variables were assessed with Student’s *t*-test (normally distributed variables) or the Mann–Whitney U test (non-normally distributed variables); effect size was calculated as rank-biserial correlation coefficient.

For categorical variables, Fisher’s exact test or chi-square test was performed, as appropriate; effect size was calculated via the Phi Coefficient (φ).

Select clinically relevant variables were entered into a multivariable logistic regression model to assess independent predictors of clinical response. Due to the limited number of non-responders in the cohort, the number of predictors was restricted to minimize risk of overfitting.

Discriminatory ability of possible indicators was tested with receiver operating characteristic [ROC] curves.

In all the analyses, a two-tailed probability value of *p* < 0.05 was considered statistically significant.

## 3. Results

### 3.1. Sample Characteristics

In total, 55 patients who had undergone biological therapy from March 2018 to March 2023 at the Department of Gastroenterology were included in this study—33 men and 22 women; in total, 57 courses of therapy; *n* = 51 (89.5%) received IFX and *n* = 6 (10.5%) received ADA.

A single-center retrospective cohort study was performed. Adult patients with Crohn’s disease who initiated anti-TNF therapy between March 2018 and March 2023 were included. The primary endpoint was clinical response at week 14 defined as a CDAI decrease of ≥70 or 25% from baseline.

In total, 55 patients received 57 courses of anti-TNF therapy between March 2018 and March 2023. Infliximab was used in 51 courses (89.5%), and adalimumab was used in six courses (10.5%).

Overall, 57 treatment courses were included. Median age at initiation of treatment was 34 years (27–43 years). A total of 27 (47.4%) patients were biologically naive, while 30 (52.6%) were biologically positive, meaning they received biological treatment as part of their treatment in the past. Clinical, laboratory and demographic characteristics in the assessed cohort were generally similar between non-responders and responders and are outlined in Table 1.

Among the assessed baseline parameters, only serum albumin differed significantly between the two groups (38.4 g/L vs. 28.4 g/L, *p* = 0.047). Albumin-based indices displayed a similar signal but failed to reach the significance threshold. Additionally, NLR and CDAI exhibited a non-significant disparity between the two groups.

The group was then subdivided into bionaïves/biopositives to further investigate whether any pre-treatment parameter outliers in regard to clinical response are present, as shown in Table 2 and Table 3.

Although the number of non-responders was small (*n* = 2), the exploratory analysis nonetheless suggested differences between select laboratory parameters. Notably, a significant disparity was observed in CALLY (3.01 vs. 0.6, *p* = 0.04), while MPV and RDW showed a substantial signal (9.9 fL. vs. 11.1 fL, *p* = 0.091; 14.5% vs. 18.2%, *p* = 0.091).

Among the biopositive cohort, albumin concentrations were significantly greater among responders (39.3 g/L vs. 28.4 g/L, *p* = 0.022), derived indices displayed a trend towards significance akin to previous analyses, while MPV but not RDW varied non-significantly (9.5 fL vs. 8.7 fL, *p* = 0.152).

### 3.2. Biomarker Shift Assessment

Subsequently, pre- and post-treatment blood serum levels of assessed parameters were investigated to assess whether anti-TNF treatment resulted in any significant shift in their concentrations; BMI and the CDAI were also included in the assessment. Only parameters available at both treatment initiation and the response assessment checkpoint were included. Delta values were used with regard to the responder and non-responder groups.

The comparison in the total group is presented in Table 4. The shift was calculated as a subtraction, defined as Δ_d_ = Value_week 14_ − Value_week 0_.

Apart from a significant difference in the degree of shift in CDAI (*p* = 0.00002), the post-treatment monocyte count experienced a negative shift in the response subgroup in comparison to the no response subgroup (*p* = 0.04).

### 3.3. Independent Predictors of Clinical Response

As albumin concentration presented itself as the chief outlier between response and non-response groups, a multivariable logistic regression model including albumin, baseline CDAI and previous biologic exposure was constructed. Albumin concentration was independently associated with clinical response (OR 1.5, 95% CI 1.16–1.97; *p* = 0.002), while baseline CDAI (OR 1.03, 95% CI 0.98–1.07; *p* = 0.219) and previous biologic exposure (OR 8.14, 95% CI 0.46–144.13; *p* = 0.153) were not. The model demonstrated acceptable overall goodness-of-fit (Hosmer–Lemeshow = 2.22, *p* = 0.973).

### 3.4. ROC Analysis

Further, ROC analysis was carried out in order to identify any markers with potential value in regard to treatment response. Markers we identified as most promising were emphasized in the analysis. Furthermore, unremarkable results were omitted, hence the specific division of subgroups.

Results of ROC analysis are reported in Table 5.

Among the assessed variables, albumin and CALLY were identified as stimulants, and CRP/Albumin, MPV and RDW were identified as destimulants when binary outcome was set to clinical response as defined by sufficient reduction in CDAI.

In the total cohort, albumin and albumin-derived indices showed good discriminative performance for clinical response (albumin AUC 0.93, CALLY 0.81, CRP/albumin ratio 0.83). In biopositive patients, albumin and CALLY reached AUC values of 1.0 and 0.88, respectively. In bionaïve patients, MPV and RDW demonstrated good predictive value (AUC 0.87 and 0.88).

In the assessed sample, mean albumin concentrations as well as CALLY and CRP/Albumin ratio proved to be valid predictors of clinical response among the total and biopositive groups, with higher CALLY and lower CRP/albumin ratio values acting as positive predictors for better likelihood of a favorable clinical outcome.

On the contrary, among the bionaïve sample, the inflammatory markers MPV and RDW presented as predictors instead, with lower MPV/RDW values coinciding with better response rates. This is contrary to most prior research, where higher MPV is associated with favorable outcomes.

Furthermore, in the analyzed biopositive subgroup (and to a significant extent in the complete group), CALLY index and CRP/albumin ratio exhibited an almost perfect inverse correlation (Spearman’s R = −0.96, *p* < 0.001), suggesting a strong redundancy of information between the two markers. For this reason, the ROC analyses appear exactly the same, with CALLY set as stimulant and CRP/albumin as destimulant.

**Figure 1:**
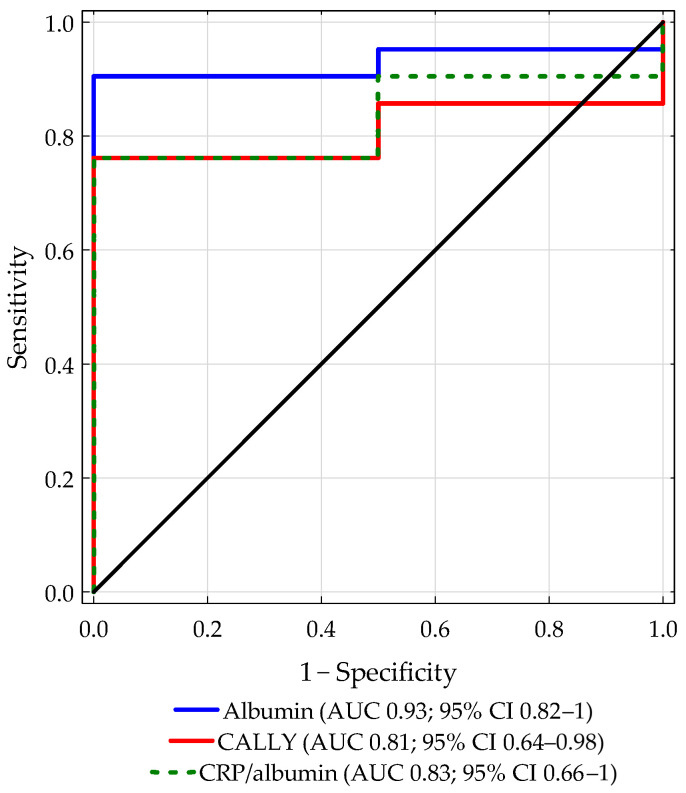
Combined receiver operating characteristic for clinical response at week 14—total group.

**Figure 2:**
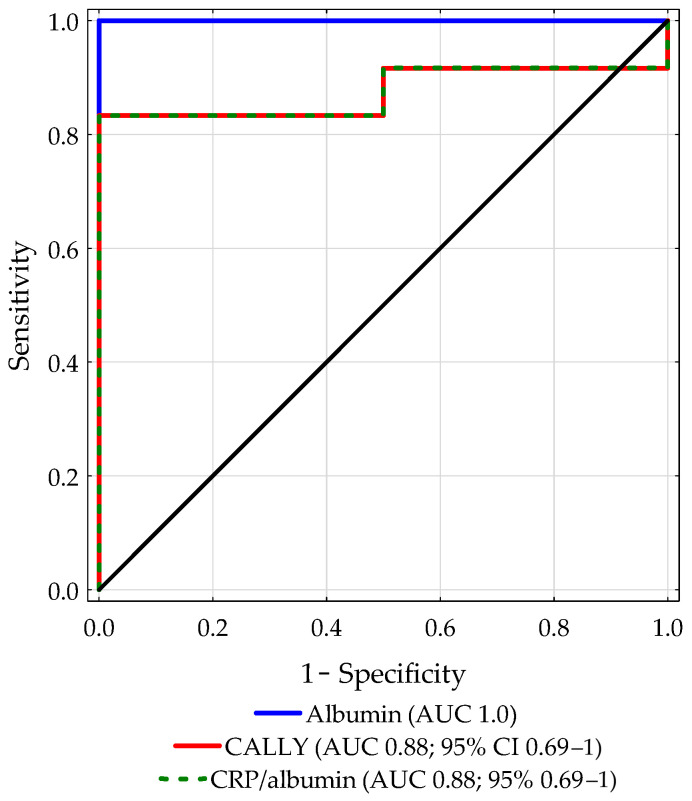
Combined receiver characteristic for clinical response at week 14—biopositive group.

**Figure 3:**
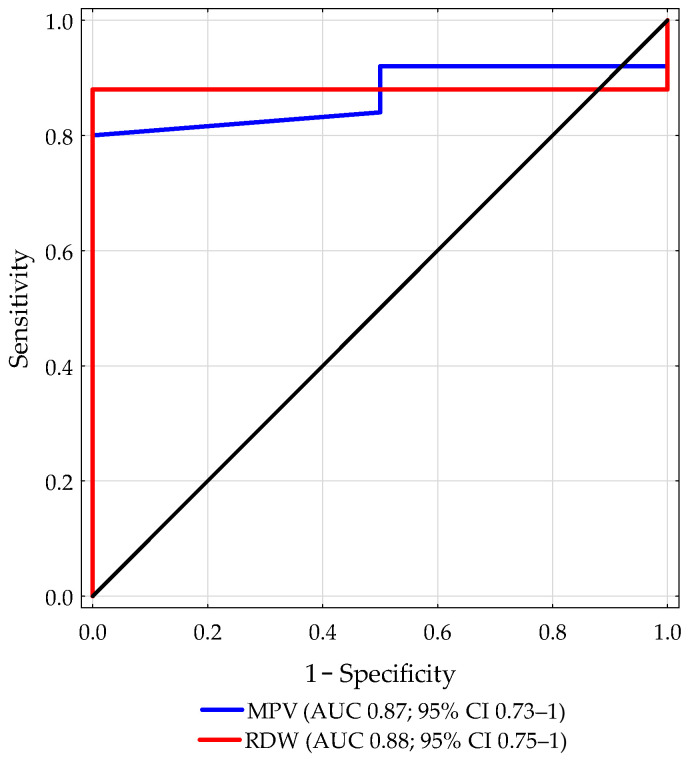
Combined receiver characteristic for clinical response at week 14—bionaïve group.

Analyses are displayed graphically depending on group division—Figure 1, Figure 2 and Figure 3.

## 4. Discussion

Our single-center retrospective cohort suggests that very simple, routinely available laboratory parameters could potentially predict early clinical response to anti-TNFs in Crohn’s disease. The field of precision medicine and individualized therapy is subject to prominent discourse, particularly in the field of gastroenterology as the molecular mechanisms responsible for the development of IBD are likely varied, often precluding uniform treatment approaches [12]. Recent advances in artificial intelligence and other data-driven methods support this shift toward personalized medicine and custom treatment, although extensive validation and clinical control remain paramount before widespread adoption of such methods will become feasible [13].

Baseline serum albumin emerged as the only independent predictor of clinical response in the whole cohort, with excellent discriminative performance (AUC 0.93; optimal cut-off ~31 g/L) and even perfect separation between responders and non-responders in biopositive patients (AUC 1.0 at ~32 g/L). Additionally, multivariable analysis displayed that the relationship between albumin and clinical response remained remarkable after adjustment for baseline CDAI and previous biologic exposure. Two composite indices incorporating albumin—the CALLY index and the CRP/albumin ratio—also demonstrated good to very good accuracy (AUC 0.81–0.88) in both the overall population and the biopositive subgroup. In biologic-naïve patients, red cell distribution width (RDW) and mean platelet volume (MPV) showed similarly strong predictive value (AUC ~0.87–0.88), with clearly defined cut-offs. Taken together, these observations indicate that albumin and simple hematologic indices may potentially be useful tools for risk stratification before anti-TNF induction in Crohn’s disease. The models achieved overall high discriminative performance; however, interpretation is limited by the small number of non-responders (*n* = 7), increasing the risk of overfitting. As such, the analyses should be interpreted with caution, especially in the bionaïve or biopositive subgroups.

Albumin is a negative acute-phase reactant and a composite marker reflecting systemic inflammation, nutritional status and chronic disease burden in IBD. It has been associated with disease activity, postoperative outcomes and long-term prognosis across multiple IBD cohorts [14,15,16,17]. Our findings are consistent with this broader literature and extend it by showing that a single baseline measurement of albumin may effectively discriminate future responders from non-responders to anti-TNF therapy in a real-world Crohn’s disease population.

Several previous studies have also identified low baseline albumin as a predictor of unfavorable outcomes under biological treatment. In a large Asian cohort of patients with Crohn’s disease and ulcerative colitis treated with anti-TNF agents, Kumar et al. found that serum albumin was the strongest independent predictor of both primary non-response and secondary loss of response, particularly in settings without routine therapeutic drug monitoring [18]. Our data—in a smaller but clinically heterogeneous European cohort treated mainly with infliximab and, to a lesser extent, adalimumab—are directionally in line with these observations and support the concept that albumin is a robust, generalizable marker of biological treatment success in IBD.

The mechanistic underpinning of this association likely relates to pharmacokinetics. Population pharmacokinetic modeling has demonstrated that low albumin is one of the key patient factors associated with increased infliximab clearance and a shorter effective half-life, along with high body weight and anti-drug antibody formation. Patients with hypoalbuminemia achieve lower trough concentrations for a given mg/kg dose, predisposing them to primary non-response and early loss of response [19]. Our results therefore complement pharmacokinetic data by showing that a simple pre-treatment albumin measurement—without direct drug level assessment—can act as an inexpensive surrogate for unfavorable exposure profiles in everyday practice.

Beyond absolute albumin concentrations, composite indices that integrate albumin with inflammatory markers have gained increasing attention. Several studies have shown that the CRP/albumin ratio correlates with clinical and endoscopic disease activity in Crohn’s disease and ulcerative colitis and can outperform CRP alone as a marker of active inflammation [9,20,21]. In Crohn’s disease, Qin et al. demonstrated that both serum albumin and the CRP/albumin ratio were closely associated with disease activity indices and endoscopic severity [7]. Chen et al. subsequently confirmed in a mixed IBD cohort that higher CRP/albumin ratios were linked with more active disease and worse outcomes [22]. More recently, Nguyen et al. reported that the CRP/albumin ratio has practical utility in day-to-day management of IBD, including monitoring biological therapy and flagging patients at risk of treatment failure [21].

In this context, our study adds two important points. First, it shows that the CRP/albumin ratio, with an optimal cut-off around 1.35, is not only a marker of disease activity but also a promising predictor of clinical response to anti-TNF induction in Crohn’s disease, including in biopositive patients. Second, to our knowledge, it is among the first reports to evaluate the CALLY index (which combines CRP, albumin and lymphocyte count) specifically as a predictor of anti-TNF response in IBD. While the CALLY index has been used in other inflammatory and oncological conditions, its application in IBD has been much less explored. Our data suggest that values ≥0.7–0.8 may identify patients with a particularly high probability of achieving early response under anti-TNF therapy, especially in the biopositive cohort setting. Because all three components of the CALLY index are part of routine laboratory panels, this metric could be implemented at virtually no additional cost.

We also observed high predictive value for two hematological indices—RDW and MPV—in biologic-naïve patients. Elevated RDW has been repeatedly associated with disease activity in Crohn’s disease and other forms of IBD, reflecting the combined effects of chronic inflammation, iron deficiency and impaired erythropoiesis [23,24]. In a prospective cohort, Hu et al. reported that RDW discriminated active from inactive Crohn’s disease with a performance comparable to, or better than, classical inflammatory markers, such as ESR and leukocyte count [25]. Meta-analyses and narrative reviews have further highlighted RDW as a low-cost, widely available marker that tracks IBD activity and may contribute prognostic information in various autoimmune conditions [23,26,27]. Our findings extend this evidence by indicating that, in biologic-naïve Crohn’s disease, RDW values below approximately 17.7% are strongly associated with achieving meaningful clinical response during anti-TNF induction.

Mean platelet volume, in turn, is a surrogate of platelet turnover and activation. In IBD, platelet counts are often elevated, while MPV tends to be reduced, presumably due to consumption of larger, more reactive platelets at sites of intestinal inflammation. A recent systematic review and meta-analysis by Bambo et al. showed that MPV is significantly lower in IBD patients than in healthy controls, with the largest differences observed in Crohn’s disease [28]. A more recent meta-analysis of platelet parameters in IBD by Xu et al. confirmed that altered platelet indices, including MPV, are characteristic of active disease, although results across individual studies remain heterogeneous [29]. In our biologic-naïve subgroup, MPV displayed very good discrimination for treatment response (AUC ~0.87, cut-off >10.6 fL), suggesting that patients with relatively preserved platelet indices may have a more favorable course under first-line anti-TNF therapy. These findings should, however, be interpreted cautiously, given the limited sample size and the known variability of MPV measurements between laboratories.

Despite promising signals, several limitations of our work must be acknowledged. This study is retrospective and single-center, with a modest sample size, especially among the non-responders, which increases the risk of both type I and type II error and may inflate AUC estimates. Additionally, bootstrapping, cross-validation or external validation was not performed, further underlining the necessity of cautious interpretation. The cohort is heterogeneous with respect to prior biological exposure (bionaïve versus biopositive patients) and largely dominated by IFX, with a handful of patients receiving ADA, making the analyses primarily applicable to those treated with IFX. In addition, other biologics, such as vedolizumab or ustekinumab, were not evaluated in this study. Therapeutic drug monitoring data (trough levels and anti-drug antibodies) were not available, preventing direct assessment of how albumin and the studied indices relate to infliximab or adalimumab exposure. Moreover, our endpoint was clinical response at week 14, without systematic endoscopic or radiologic confirmation of mucosal healing. Finally, laboratory parameters were assessed only at baseline; dynamic changes during induction and maintenance were not captured and might provide additional prognostic information. Overall, the outlined observations require verification in larger, more varied cohorts, especially in the context of responders and non-responders.

Nonetheless, the strengths of this study lie in the use of routinely available, inexpensive laboratory markers and in the inclusion of a “real-life” Crohn’s disease population treated under standard clinical conditions. Our results support the notion that baseline albumin—alone or as part of composite indices such as the CRP/albumin ratio or CALLY—can meaningfully inform the probability of success of anti-TNF induction. In settings where therapeutic drug monitoring is unavailable or limited, these markers may help identify patients at higher risk of non-response who might benefit from closer follow-up, early dose optimization, or consideration of alternative therapeutic strategies. Future prospective, multicenter studies with larger cohorts, systematic drug level measurements and hard outcomes (including endoscopic healing and surgery) are needed to validate the proposed cut-offs and to define how best to integrate albumin-based indices, RDW and MPV into practical treatment algorithms.

## 5. Conclusions

This study demonstrates that simple, readily available serum-based markers can serve as early treatment response predictors in CD patients receiving anti-TNF agents. Especially serum albumin and albumin-derived markers as well as certain hematological parameters appear promising in regard to early treatment outcomes. The presented results facilitate further research in this field.

## Figures and Tables

**Table 1:** Baseline clinical, laboratory and demographic characteristics.

Variable	Response (*n* = 50)	No Response (*n* = 7)	*p*	Effect Size
Sex, n (%)				
Female	19 (33.33%)	3 (5.2%)	0.556	0.033
Male	31 (54.3%)	4 (7%)		
Age (years)	34.5 (26–44)	34 (28–35)	0.618	0.12
Disease duration (years)	7.0 (3–10)	9.0 (5–11)	0.451	0.18
BMI (kg/m^2^)	23.2 (20.0–26.1)	24.2 (19.3–27.6)	0.551	0.142
CRP (mg/L)	11.5 (3.5–32.9)	13.2 (3.2–77.7)	0.535	0.149
Albumin (g/L)	38.4 (35.0–41.0)	28.4 (26.7–30.1)	**0.047**	**0.643**
Monocytes (×10^3^/µL)	0.63 (0.40–0.80)	0.55 (0.39–0.87)	0.752	0.077
MPV (fL)	9.9 (9.3–10.5)	9.3 (8.5–10.9)	0.670	0.102
RDW (%)	14.1 (13.1–16.1)	14.1 (3.5–17.8)	0.715	0.089
CRP/Albumin ratio	0.37 (0.10–1.35)	2.73 (1.72–3.73)	0.141	0.312
CALLY index	2.21 (0.71–11.22)	0.47 (0.33–0.60)	0.172	0.287
PLR	255 (167–413)	262 (200–540)	0.836	0.051
NLR	3.42 (2.38–7.05)	5.56 (2.90–7.54)	0.220	0.291
MLR	0.52 (0.41–0.71)	0.46 (0.31–0.73)	0.893	0.034
SII	1177 (735–2440)	1473 (967–2760)	0.362	0.217
CDAI (on admission)	342.5 (315–408)	312 (296–346)	0.137	0.354

Bold means significant values.

**Table 2:** Bionaïve sample—characteristics.

Variable	Response (n = 25)	No Response (*n* = 2)	*p*
Sex, n (%)			
Female	12 (44.44%)	1 (3.7%)	0.741
Male	13 (48.1%)	1 (3.7%)	
Age (years)	35 (27–44)	28.5 (22–35)	0.319
Disease duration (years)	4 (1–8)	5 (1–9)	0.963
BMI (kg/m^2^)	23.44 (20.93–26.54)	26.49 (25.40–27.58)	0.279
CRP (mg/L)	9.4 (2.4–15.5)	8.2 (3.2–13.2)	1.000
Albumin (g/L)	35.8 (31.0–41.3)	27.3 (26.6–28)	0.145
Monocytes (×10^3^/µL)	0.62 (0.46–0.71)	0.52 (0.48–0.55)	0.689
MPV (fL)	9.9 (9.4–10.6)	11.1 (10.9–11.3)	**0.091**
RDW (%)	14.5 (13.3–16.4)	18.2 (17.8–18.6)	**0.091**
CRP/Albumin ratio	0.30 (0.10–1.98)	0.31 (0.11–0.5)	0.91
CALLY index	3.01 (0.56–15.12)	0.6 (0.35–0.99)	**0.04**
PLR	241.6 (186.63–340)	216.91 (141.71–292.10)	0.569
NLR	3.11 (2.45–4.52)	2.84 (2.77–2.90)	0.627
MLR	0.49 (0.31–0.64)	0.37 (0.31–0.42)	0.513
SII	1140 (895.05–1664.95)	827.8 (688.73–966.87)	0.319
CDAI (on admission)	340 (319–392)	317 (296–338)	0.462

Bold means significant values.

**Table 3:** Biopositive sample—characteristics.

Variable	Response (*n* = 25)	No Response (*n* = 5)	*p*
Sex, n (%)			
Female	7 (28.0%)	0 (0.0%)	0.236
Male	18 (72.0%)	5 (100.0%)	
Age (years)	31 (17–32)	34 (33–35)	0.914
Disease duration (years)	8 (5–11)	10 (9–11)	0.516
BMI (kg/m^2^)	24.09 (20.42–28.73)	23.97 (19.33–24.21)	0.872
CRP (mg/L)	19.3 (6.3–54.2)	51.9 (3.7–77.7)	0.589
Albumin (g/L)	39.3 (35.15–40.85)	28.4 (26.7–30.1)	**0.022**
Monocytes (×10^3^/µL)	0.66 (0.39–0.82)	0.59 (0.39–0.87)	0.914
MPV (fL)	9.5 (9.2–10.1)	8.7 (8.5–9.3)	0.152
RDW (%)	13.8 (13.1–15.6)	14.0 (13.5–14.1)	1.000
CRP/Albumin ratio	0.39 (0.12–1.25)	2.72 (1.72–3.73)	0.113
CALLY index	1.90 (0.95–8.13)	0.47 (0.33–0.60)	0.132
PLR	325.33 (157.14–415.85)	262.22 (218.32–540.35)	0.626
NLR	3.42 (2.35–9.12)	6.17 (5.55–7.54)	0.229
MLR	0.54 (0.32–0.80)	0.66 (0.46–0.73)	0.746
SII	1193.14 (614.84–3110.60)	2323.5 (1473.66–2759.82)	0.355
CDAI (on admission)	345 (315–410)	312 (311–346)	0.188

Bold means significant values.

**Table 4:** Comparison of degree of shift in select parameters.

Variable	Response	No Response	*p*
BMI (kg/m^2^)	0.92 (0–2.03)	1.19 (0–1.86)	0.559
CRP (mg/L)	−2.5 (−16.2–0.1)	−3.3 (−25.6–13.7)	0.972
Monocytes (×10^3^/µL)	−0.03 (−0.14–0.12)	0.18 (0.02–0.49)	**0.040**
MPV (fL)	0.45 (0.1–0.9)	0.40 (0.1–0.7)	0.858
RDW (%)	−0.85 (−2.9–0.1)	3.04 (−2.7–1.2)	0.332
PLR	−71.51 (−239.97–−4.49)	−57.69 (−97.67–−0.68)	0.659
NLR	−0.82 (−3.65–0.22)	0.76 (−2.37–3.87)	0.113
CDAI (on admission)	−213 (−259–−180)	−68 (−76–−37)	**0.00002**
SII	−384.16 (−1416.03–24.78)	−420.48 (−831.11–1009.31)	0.511

Bold means significant values.

**Table 5:** CaptionReceiver Operating Characteristic analysis of individual markers for short-term treatment response.

Variable	Sample	AUC (95% CI)	Sensitivity (%)	Specificity (%)	Cut-off	*p*
Albumin (g/L)	Total	0.93 (0.82–1)	90.5	100.0	>31.0	**<0.001**
	Biopositive	1.00 (1)	100.0	100.0	>32.2	**<0.001**
CALLY	Total	0.81 (0.64–0.98)	76.2	100.0	>0.71	**0.0005**
	Biopositive	0.88 (0.69–1)	83.33	100.0	>0.77	**0.0001**
CRP/Albumin ratio	Total	0.83 (0.66–1)	76.2	100.0	<1.35	**0.0002**
	Biopositive	0.88 (0.69–1)	83.33	100.0	<1.35	**0.0001**
MPV (fL)	Bionaïve	0.87 (0.73–1)	80.0	100.0	<10.6	**<0.001**
RDW (%)	Bionaïve	0.88 (0.75–1)	80.0	100.0	<17.7	**<0.001**

Bold means significant values.

## Data Availability

The original contributions presented in this study are included in the article. Further inquiries can be directed to the corresponding author.

## Abbreviations

The following abbreviations are used in this manuscript:

IFX	Infliximab
ADA	Adalimumab
CD	Crohn’s disease
anti-TNF	Anti-tumor necrosis factor
ROC	Receiver operating characteristic
AUC	Area under curve
CALLY	C-reactive protein–albumin–lymphocyte index
MPV	Mean platelet volume
RDW	Red cell distribution width
IBD	Inflammatory bowel disease
CDAI	Crohn’s disease activity index
ECCO	European’s Crohn’s and Colitis Organization
BMI	Body mass index
PLR	Platelet–lymphocyte ratio
NLR	Neutrophil–lymphocyte ratio
MLR	Monocyte–lymphocyte ratio
SII	Systemic immune-inflammation index
CRP	C-reactive protein
